# The first case of *Mycobacterium vaccae* sepsis in a non-Hodgkin lymphoma patient: biological understandings and clinical consequencies

**DOI:** 10.1099/acmi.0.000161

**Published:** 2020-08-25

**Authors:** Pierluigi Congedo, Angelo Gardellini, Lucia Corich, Angela Papa, Mauro Turrini

**Affiliations:** ^1^​ Operative Unit of Chemical-Clinical and Microbiological Analysis, Ospedale Valduce, Como, Italy; ^2^​ Clinical Pathology Laboratory Vimercate Hospital, Italy; ^3^​ Division of Hematology, Department of Medicin, Ospedale Valduce, Como, Italy

**Keywords:** CRBSI, Lymphoma, MALDI-TOF, *Mycobaterium vaccae*, sepsis

## Abstract

*
Mycobacterium vaccae* is a rapidly growing nonpathogenic species of the *Mycobacteriaceae* family of bacteria that can cause pulmonary and disseminated disease in particular in immunocompromised individuals. Here we describe a first case of matrix-assisted laser desorption/ionization-time of flight (MALDI-TOF) mass-spectrometry (MS) identification of this pathogen in a patient with non-Hodgkin’s lymphoma during chemoimmunotherapy salvage treatment, and its impact on clinical decision making.

## Introduction

*Mycobacterium vaccae* is known as a nonpathogenic species of the *Mycobacteriaceae* family. Clinicians have become increasingly conscious that species of mycobacteria previously considered nonpathogenic can cause serious diseases in immunocompromised patients. In particular, in recent years there is an increasing interest in the role of nontuberculous mycobacteria (NTM) as pathogens causing pulmonary and disseminated disease in both immunocompetent and immunocompromised individuals [1]. *M. vaccae *, a yellow-pigmented, rapidly growing mycobacterium (RGM), belonging to the NTM family, was first isolated, described and named in 1962 [2]. Several strains of these bacteria were isolated from the environment in which cattle live, including the soil and water, as well as from bovine lactic ducts, skin nodules and milk products. Most infections are due to accidental inoculation from trauma, surgery, injection or aspiration. Four cases of invasive disease in humans (cutaneous and pulmonary) were for the first time reported in 1996 [3]. Until now no evidence of person-to-person transmission has been reported [4].

Here, we describe the case of a follicular non-Hodgkin lymphoma (NHL) patient that experimented a rare infectious complication by *M. vaccae* during a salvage chemotherapy course.

To our knowledge, this is the first report of documented *M. vaccae* sepsis.

## Case report

**Fig. 1.**
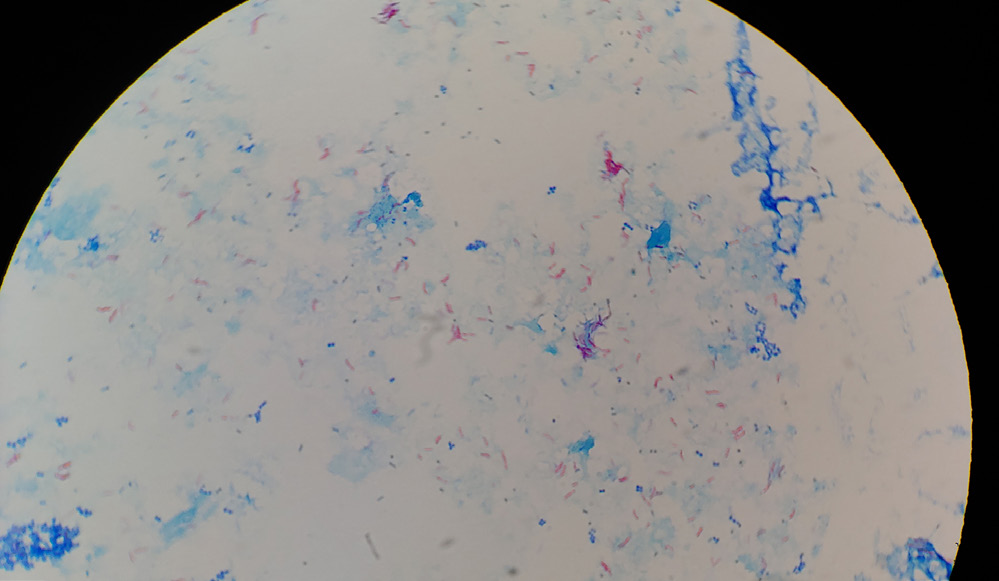
BAAR visualization by Ziehl-Neelsen stain.

**Fig. 2.**
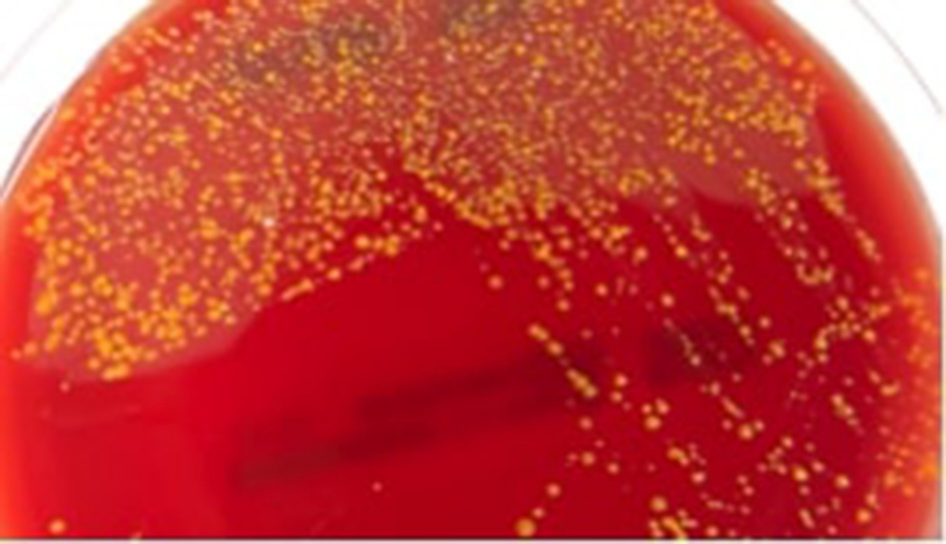
Colonies of *M. vaccae* on a blood agar plate.

**Fig. 3.**
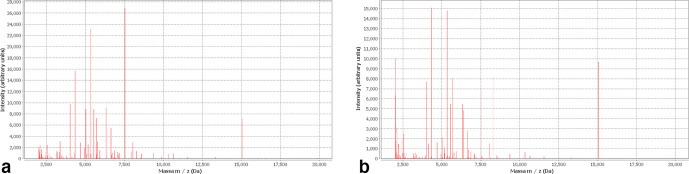
Identification of *M. vaccae* by MALDI-TOF MS.

A 49-year-old caucasian female presented at Hematology Division of Valduce Hospital (Como, Italy) on March 2012 with front nodular skin lesions (1.3 up to 2.5 cm). She underwent biopsy at this level and a diagnosis of follicular lymphoma was performed. During the visits she did not report classic constitutional symptoms like fevers, chills, night sweats, fatigue or weight loss. Her past medical history was not significant for excessive alcohol consumption, for tobacco use, insulin-dependent type 2 diabetes mellitus, hypertension, particular infectious diseases. Laboratory data revealed thrombocytopenia with platelet count ranging from 80×10^9^ l^−1^ to 90×10^9^ l^−1^, and leuocopenia (white blood cells 3.8×10^9^ l^−1^) with lymphopoenia (0.500×10^9^ l^−1^); normal haemoglobin value (13.6 g dl^−1^), and normal assessment of renal and hepatic function were detected. Lactate dehydrogenase (LDH) was in normal range (164 U l^−1^ with laboratory normal range=100–190 U l^−1^). Human immunodeficiency virus (HIV) and hepatitis B serologies were unremarkable. Computed tomography (CT) revealed multiple lymphoadenopaties (maximum size 2.8 mm) and splenomegaly (larger diameter 16 cm). Positron emission tomography/computed tomografy (PET/CT) showed right hypermetabolic uptake at these levels. Finally, bone marrow biopsy was negative for lymphoma localization. From June to November 2012, she received a total of six cycles of chemo-immunotherapy according to rituximab, cyclophosphamide, hydroxydaunorubicin, oncovin and prednisone schedule (R-CHOP), obtaining first complete remission (CT scan and PET negativity). A maintenance regimen with monoclonal anti-CD20 antibody every 2 months was started [5, 6]. Regular follow-up was performed and on July 2017 she experimented relapse with appearance of new cutaneous lesions. The biopsy was done and the histological evaluation confirmed follicular NHL relapse. CT scan and PET were congruent. Since January 2018 we started salvage treatment according to rituximab, ifosfamide, carboplatin, etoposide phosphate (R-ICE) regimen with the attempt to mobilize and collect peripheral stem cell to perform autologous transplantation. The patient failed both this and plerixafor plus granulocyte colony-stimulating factor (G-CSF) mobilization, resulting a true poor mobilizer. For this reason we decided to repeat two additional R-ICE cycles as consolidation. Before starting the third drug cycle, the patient developed fever with chills. The blood test confirmed the presence of leucopenia with severe lymphopenia; on the contrary neutrophils were in normal range (2.000×10^9^ l^−1^). Blood cultures by peripheral venipuncture and from peripherally inserted central catheter (PICC) were collected: bottles were incubated in BD-Bactec 9120 Blood Culture System instrumentation (Becton Dickinson) and 3 days later samples resulted positive. Difference in positivity time (2 h) between PICC and peripheral venipuncture and the isolation of the same micro-organism suggested a catheter-related bloodstream infection (CRBSI), also called catheter-related sepsis. Gram stain showed bacilli characterized by dubious features that were confirmed by acid fast stain, Ziehl-Neelsen staining (Fig. 1), which highlights the bacilli-acid-alcohol-resistant (BAAR). Blood samples were inoculated on Blood Agar plates, incubated at 37 °C, 0.5 % CO_2_ (carbon dioxide) and the day after the colonies (Fig. 2) were analysed by matrix-assisted laser desorption/ionization-time of flight (MALDI-TOF) mass spectrometry (MS) (VITEKMS Biomerieux) for identification, resulting *M, vaccae* (Fig. 3). Meanwhile an empiric antibiotic treatment with piperacillin/tazobactam (4.5 g three times a day) had been started, afterwards replaced by levofloxacin (750 mg once daily) because of liver toxicity. In order to confirm MALDI-TOF identification, a 16S ribosomal RNA (rRNA) sequencing analysis was performed. Antibiotic susceptibility was tested using Sensititre RAPMYCOI (Thermoscientifìc) following the manifacturer’s protocol. Specific clinical breakpoints for antimicrobial agents were not available. The intravenous catheter was removed and blood cultures have been repeated using specific bottles for Mycobacteria: samples were negative. After catheter removal, chest x-ray did not reveal pneumonia, furthermore a rapid reduction of fever and a decrease of systemic inflammation biomarkers were observed.

## Discussion

*M. vaccae* is a RGM species previously not considered a human pathogen. Despite this, some reports indicate it as lung and cutaneous pathogen [3]. Thanks to its rapid growth characteristic, it can be cultivated even on non-selective media for mycobacteria and this has allowed its unexpected identification by using the common procedures of sample preparation for MALDI-TOF analysis used for non-mycobacteria. Indeed *M. vaccae* was identified by direct apposition of the colony, grown on Columbia Agar media after overnight incubation in CO_2_, to which one microlitre of matrix consisting of a saturated solution of alpha cyano hidroxycinnamic acid dissolved in 50 % acetonitrile and 2.5 % trifluoroacetic acid, was added. So, no pretreatment procedure was necessary to analyse our sample. However, it is well known that the process of inactivation and extraction of proteins has to be applied in order to identify mycobacteria by MS [7]. When the identification without specific pretreatment is performed, a final confirmation with an alternative method, i.e. 16S rRNA sequencing, is required. In our case, this second method confirmed a *M. vaccae* CRBSI.

This work highlights the usefulness of the MALDI-TOF method in routine laboratory practice for the identification of mycobacteria due to its ability to identify RGM even without pretreatments.

The use of MALDI-TOF for identification of NTM has been crucial for assessing the clinical significance of the micro-organism isolated from several clinical samples, such as sputum, bronchoalveolar lavage, gastric lavage, urine, pus, peritoneal fluid and biopsy and grown in solid or liquid media. Indeed, several NTM species can cause serious pulmonary disease in children or skin disease and disseminated infections in immunocompromised patients. MALDI-TOF has showed to be capable of performing reliable and accurate identification of these species, allowing prompt initiation of appropriate antimicrobial therapy, optimizating the patient management [8, 9].

Besides, over the past 10 years, MALDI-TOF has radically changed the microbiological diagnostics reducing the identification time of microorganisms with a significant public health impact. The processing method is faster than molecular methods resulting in greater cost-effectiveness advantage and ensuring identification at the species level.

Considering the absence of specific clinical breakpoints for this pathogen, the patient was successfully treated only empirically. On the other hand, as the septic complication by this agent is a symptom of profound immunodepression, the fast identification was helpful to immediately discontinue any immunochemioterapic treatment. We continued only the patient follow-up. According to our opinion, this approach was the most safe for the patient.
